# Effect of an Insulation Device in Preventing Hypothermia during Magnetic Resonance Imaging Examinations for Dogs and Cats under General Anesthesia

**DOI:** 10.3390/ani11082378

**Published:** 2021-08-12

**Authors:** Eri Onozawa, Daigo Azakami, Seri Seki, Yuji Hamamoto, Katsumi Ishioka

**Affiliations:** 1 School of Veterinary Nursing and Technology, Faculty of Veterinary Science, Nippon Veterinary and Life Science University, Tokyo 180-8602, Japan; seki.seri@nvlu.ac.jp (S.S.); katsumi@nvlu.ac.jp (K.I.); 2 Laboratory of Veterinary Clinical Oncology, Faculty of Agriculture, Tokyo University of Agriculture and Technology, Tokyo 183-8509, Japan; azakami@go.tuat.ac.jp; 3 Veterinary Medical Teaching Hospital, Nippon Veterinary and Life Science University, Tokyo 180-8602, Japan; y-hamamoto@nvlu.ac.jp

**Keywords:** cat, dog, heat insulating, hypothermia, MRI examination

## Abstract

**Simple Summary:**

Magnetic resonance imaging examinations require general anesthesia, and it is difficult to prevent a decrease in body temperature because a machine for warming the body cannot be placed in the magnetic resonance imaging room, which must have a low room temperature. In this study, we created a heat insulating device that does not affect magnetic resonance imaging and examined the effectiveness of this device for dogs and cats undergoing magnetic resonance imaging examinations. In the dogs and cats wearing bubble wrap and down cloth blanket, the decrease in body temperature was minimal. The heat insulating device developed in this study protected the animals from the cold air and prevented heat loss from the body surface, minimizing a decrease in body temperature. The results obtained in this study suggest that dogs and cats requiring magnetic resonance imaging can be protected from hypothermia due to general anesthesia without the need for special machinery.

**Abstract:**

Dogs and cats under general anesthesia may develop hypothermia. When performing a magnetic resonance imaging (MRI) examination, it is not possible to place a magnetic material in the MRI room, and MRI equipment requires a low room temperature. This study investigated the effectiveness of a heat insulating device that prevented hypothermia during MRI examinations in dogs and cats. The animals that underwent MRI examinations under general anesthesia were divided into control groups (no covering) and heat insulating groups (wearing bubble wrap and down cloth blankets), and their body temperatures were measured before and after the MRI examinations. The changes in body temperatures were as follows: control dogs (*n* = 17), median of −1.0 (from −2.5 to 0.3) °C; heat insulated dogs (*n* = 7), −0.3 (from −0.8 to 0.2) °C; control cats (*n* = 14), −1.85 (from −2.7 to −0.6) °C; and heat insulated cats (*n* = 12), −0.8 (from −1.5 to −0.1) °C. These results revealed that the bubble wrap and down cloth blanket significantly prevented hypothermia and heat loss from the body surface during MRI examinations of dogs and cats.

## 1. Introduction

Hypothermia is a very common complication in dogs and cats under anesthesia and is reported to occur in approximately 40% of anesthetized animals [1,2,3,4]. Hypothermia can cause various adverse events such as delayed extubation time, organ dysfunction, decreased immune function, abnormal blood coagulation, and decreased metabolism [5,6]. The hypothalamus, which is the thermoregulatory center, is suppressed by anesthetics, causing hypothermia within minutes of the induction of general anesthesia in animals. During anesthesia, heat convection, radiation, conduction, and evaporation dissipate the body heat into the environment and decrease heat production, resulting in a decrease in the body temperature [6]; therefore, it is important to warm the environment around the animal in order to maintain the body temperature.

In human medicine, the use of a warm air heating device for patients undergoing surgery prevents hypothermia [7,8,9,10] and has a heat retaining effect on patients undergoing surgery. When aluminum sheets and cushioning materials used for packaging were used, both were effective in preventing hypothermia. In veterinary medicine, various heating and heat insulating methods for dogs and cats under anesthesia management are being studied [11,12,13,14,15,16,17,18]. A hot water bottle is a typical heating method used in many hospitals because it can easily heat animals without a dedicated machine. Further, in veterinary medicine, as well as in human medicine, it has been clarified that hypothermia can be effectively prevented using a warm air heating device or an electric heat mat during surgery [15,16]. Heat dissipation from the skin surface can be reduced by 30% by covering with a towel, blanket, or aluminum sheet not only for heating but also for retaining heat [19]. In addition, the decrease in body temperature is suppressed by wrapping a packing cushioning material and plastic-lined pad around the body trunk (other than the surgical wound) and limbs during neutering surgery for cats [18]. The results reported on the prevention of hypothermia in veterinary medicine have often been from surgical studies [11,12,13,14,15,16,17,18].

In veterinary medicine, general anesthesia use is essential not only during surgery but also during imaging examinations such as magnetic resonance imaging (MRI). Due to the control of the instruments, the MRI room must be set to about 20 °C, which is a very cold environment for anesthetized animals. Despite the fact that hypothermia is likely to occur due to effects of anesthesia, machines such as heat mats and warm air heaters cannot be used in the MRI room. Therefore, at our veterinary hospital, we use hot water bottles made from infusion bags to warm animals, but it is difficult to prevent hypothermia at the end of the MRI exam. Since it is not possible to use a heating device that is effective during surgery, it was necessary to design a heat insulating material that does not react to magnetism. For a dog that underwent an MRI exam in 2017, an attempt was made to warm the air in the circuit by attaching a heat and moisture exchanger between the circuit hose and the tracheal tube, but the effectiveness of it was not demonstrated [20]. An MRI exam has a relatively short anesthesia time compared to surgery. However, it is important to investigate heat insulating materials that are effective in preventing a decrease in body temperature in MRI acquisition using remote monitoring and care, where a device for warming the body cannot be installed. Other than the 2017 research report, there are no reports in the veterinary field that have examined heat insulting materials to be used during an MRI exam; thus, it is necessary to obtain knowledge of effective devices that prevent temperature loss for animals undergoing MRI exams. 

In this study, we investigated methods for preventing hypothermia during MRI exams in dogs and cats. We compared animals wearing bubble wrap and down cloth blanket with animals without them.

## 2. Materials and Methods

### 2.1. Preliminary Study—Examination of Heat Insulating Materials Effective in Preventing Hypothermia

**Figure 1:**
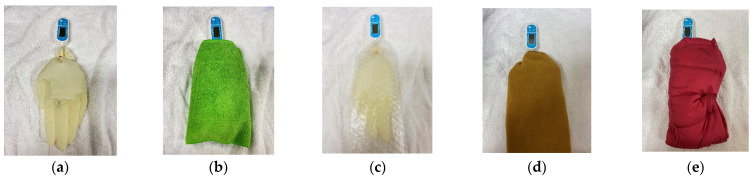
Examination of the heat insulating materials. Hot water at 39 °C was placed in a latex glove, and the temperature was measured once every 30 min from the start of measurement to 2 h later. (**a**) no cover, (**b**) bath towel, (**c**) bubble wrap, (**d**) bubble wrap and fleece, and (**e**) bubble wrap and down cloth.

We used a rubber glove (Pranatura latex glove^®^, TOP Corporation, Tokyo, Japan) as a water bottle and placed 500 mL of hot water (39 °C) with a thermometer (Waterproof Digital Thermometer^®^, GXSTWU, Ningbo, China) in it; we then wrapped it in five rubber gloves to prevent the hot water from leaking. Next, five of these glove water bottles were placed on a towel and were wrapped as follows: no cover, bath towel, bubble wrap, bubble wrap and fleece cloth, and bubble wrap and down cloth (Figure 1). The rubber gloves were covered with bubble wrap and overlaid with fleece cloth or down cloth. The temperature of the hot water was measured every 30 min for 2 h from the initial temperature of 39 °C. Most MRI exams take approximately 30 min to an hour, but sometimes two hours. Therefore, this study was conducted assuming 2 h. This process was repeated three times, and the average value and standard deviation of the temperature change over time under each condition were calculated. The room temperature in the experimental site was set to 20 °C, replicating that of an MRI room.

### 2.2. Examination of the Effect of Using a Heat Insulator to Prevent a Decrease in Body Temperature during MRI Exams in Dogs and Cats

The animals used in this study were dogs (*n* = 24) and cats (*n* = 26). The cats consisted of laboratory cats (*n* = 8) and clinical patients that visited our veterinary medical teaching hospital (*n* = 18). The owners gave permission for their use in the study, and the use of the laboratory animals was approved by the ethics committee for animal experiments, established by the Nippon Veterinary and Life Science University (2020S-5). All dogs and cats were classified as I-II by the American Society of Anesthesia (ASA).

All MR images were obtained with a 3.0-Tesla unit (Signa^®^ HDtx 3.0 T, GE Healthcare, Tokyo, Japan). An 8 channel human knee array radiofrequency coil was used. All the dogs and cats fasted for 12 h prior to the MRI and were infused with 5 mg/kg/h lactate ringer solution during scanning. A 22–24 G indwelling needle was placed in the forelimbs of all animals. After being fully oxygenated with 100% oxygen, propofol (approx. 7.0 mg/kg) (1% Propofol Injection^®^, Maruishi, Osaka, Japan) was intravenously administered and anesthesia introduced. Anesthesia was maintained with isoflurane (Escain^®^, Mylan, Tokyo, Japan) in 100% oxygen. During the MRI exam, we constantly checked SpO_2_, pulse rate, and blood pressure in all animals and took appropriate action if any abnormalities were observed. Proper control of blood pressure leads to the maintenance of cerebral blood flow. We evaluated the pain of animals by physical examination, but no pain was observed in all cases. Therefore, we did not use analgesics in this study.

**Figure 2:**
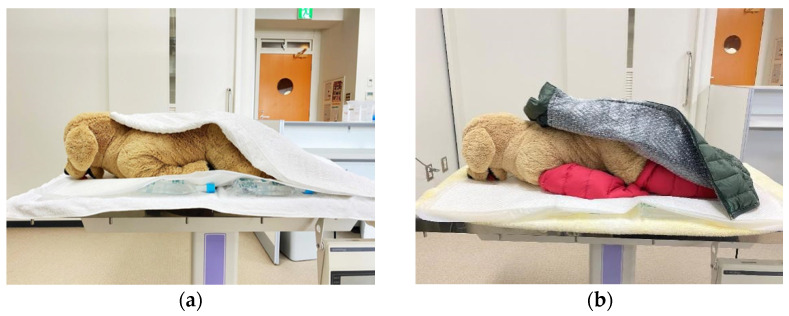
Model of the control group and the heat insulating group undergoing an MRI exam. (**a**) the control group, the animal was covered with a bath towel. (**b**) the heat insulating group, the animal was covered with bubble wrap and down cloth blanket. Both dogs were placed on hot water bottles with sheets between them.

The dogs and cats were divided into two groups; one group used the heat insulating device (heat insulation group) and the other group did not use the heat insulating device (control group). The heat insulation group consisted of 7 dogs and 12 cats, and the control group consisted of 17 dogs and 14 cats. The hot water bottle was placed beneath the sheet under the body of the animal, and the animal was covered with a bath towel in the control group (Figure 2a). Based on the preliminary study, bubble wrap and down cloth blanket was chosen for the insulation group. After installing the hot water bottle as in the control group, a heat insulation blanket made of bubble wrap and down cloth was attached to the limbs and trunk of the animals (Figure 2b).

The body temperature of the animals was measured rectally using a thermometer (Thermo Flex TF8731^®^, Astec, Japan) at the time of anesthesia induction before propofol and at the end of an MRI exam. The temperature in the MRI room was maintained at 20 °C. The dogs and cats were placed in the prone position with their heads placed in the coil. MRI was performed to detect brain abnormal lesions.

### 2.3. Statistical Analyses

For the preliminary study investigating the effectiveness of a heat insulating material in preventing a decrease in body temperature, a post hoc test (Tukey’s multiple comparison test) was performed when a significant difference was observed, using a two-way repeated measures analysis of variance (ANOVA). For the MRI exam study, a Mann–Whitney U test was performed for the evaluation of body temperature changes in the control and heat insulation groups. In all tests, a p value of less than 0.05 was considered significant (GraphPad Prism 6 analysis software, GraphPad Software, Inc., San Diego, CA, USA).

## 3. Results

### 3.1. Evaluation of the Effectiveness of the Heat Insulating Materials in Preventing Water Temperature Loss

**Figure 3:**
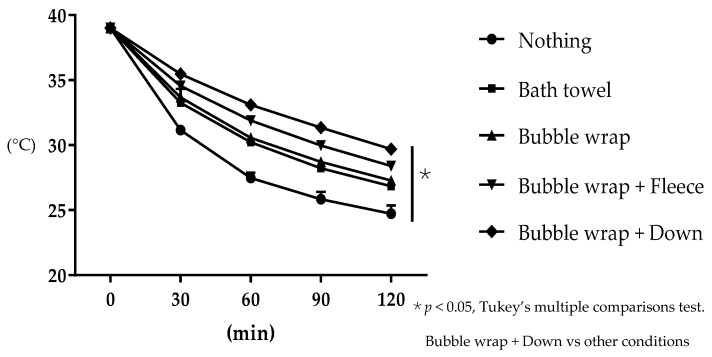
Temperature changes over time for the five heat insulators (filled circle, nothing; filled square, bath towel; triangle, bubble wrap; reversed triangle, bubble wrap and fleece; diamond, bubble wrap and down). Significant differences were observed between each condition at all measurement points (*p* < 0.05, Tukey’s multiple comparison test). The combination of bubble wrap and down is most effective in preventing temperature loss. min: minutes.

The average water temperature decreased from the start to 2 h later. The water temperatures (mean ± SD) after 30 min, 1 h, 1 h and a half, and 2 h were as follows: no cover: 31.2 ± 0.12, 27.5 ± 0.33, 25.2 ± 0.46, and 24.7 ± 0.52 °C, respectively; bath towel only: 33.3 ± 0.20, 30.2 ± 0.17, 28.2 ± 0.12, and 26.8 ± 0.05 °C, respectively; bubble wrap only: 33.7 ± 0.53, 30.6 ± 0.19, 28.7 ± 0.16, and 27.3 ± 0.05 °C, respectively; bubble wrap and fleece cloth: 34.4 ± 0.39, 31.9 ± 0.21, 30.0 ± 0.29, and 28.4 ± 0.3 °C, respectively; and bubble wrap and down cloth: 35.5 ± 0.31, 33.1 ± 0.33, 31.3 ± 0.33, and 29.4 ± 0.43 °C, respectively (Figure 3). Significant differences were observed between each condition at all measurement points (*p* < 0.05, two-way repeated measures ANOVA). In particular, when the bubble wrap and down cloth were used together, the temperature difference from the start to 2 h later was the smallest (*p* < 0.05, two-way repeated measures ANOVA). Based on these results, the heat insulating cover used in the next experiment was made of bubble wrap and down cloth.

### 3.2. Examination of the Heat Insulating Device Effectivity

The profiles of 24 dogs (17 in the control group, seven in the heat insulation group) and 26 cats (14 in the control group, 12 in the heat insulation group) were shown in Table 1 and Table 2, respectively. The MRI exam time of 24 dogs that underwent an MRI exam was 63 (range, 46–110) min in the control group and 55 (35–70) min in the heat insulating group; there was no significant difference. Additionally, the MRI exam time of 26 cats that underwent an MRI exam was 50 (38–70) min in the control group and 50 (31–62) min in the heat insulating group; there was no significant difference (*p* < 0.05, Mann–Whitney U test). Furthermore, the comparison of the body weights of each dog and cat—7.2 (2.2–24.5) kg in the dog control group and 7.0 (3.6–35.5) kg in the dog heat insulating group—showed no significant difference (*p* < 0.05, Mann–Whitney U test). The median weight of the cats was 4.4 (2.4–5.9) kg in the control group and 3.8 (3.2–6.5) kg in the heat insulating group, showing no significant difference (*p* < 0.05, Mann–Whitney U test).

**Figure 4:**
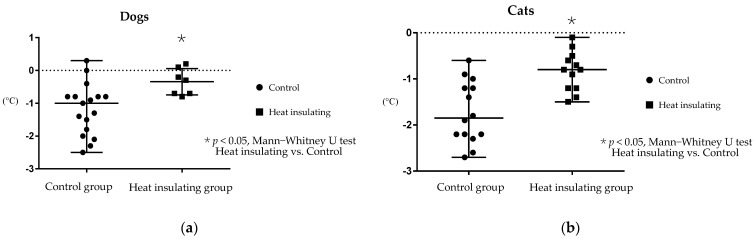
Changes in body temperature measured rectally during a magnetic resonance imaging (MRI) examination for the control group and the heat insulating group (filled circle, control; filled square, heat insulating; central large bar, median; upper and lower bars, maximum and minimum, respectively). The change in body temperature was recorded from the time of anesthesia induction to the end of the MRI examination. A decrease in body temperature was significantly prevented in the heat insulating group compared to the control group (*p* < 0.05, Mann–Whitney U test). (**a**) indicates dogs; (**b**) indicates cats.

The changes in body temperature from the time of anesthesia induction to the end of the MRI exam were as follows: dog control group, −1.0 (from −2.5 to 0.3) °C; dog heat insulating group, −0.3 (from −0.8 to 0.2) °C; cat control group, −1.85 (from −2.8 to −0.3) °C; and cat heat insulating group, −0.8 (from −1.5 to −0.1) °C (Figure 4). The mean body temperature change in the heat insulating group was significantly smaller than that in the control group for both dogs and cats (*p* < 0.05, Mann–Whitney U test). The median temperatures of control dogs before anesthesia induction and after MRI examination were 38.2 (37.5–39.8), and 37.1 (36.2–38.6), respectively. Those of control cats were 38.3 (37.7–38.7) and 36.3 (35.7–37.7), respectively. The median temperature of heat insulation group dogs before anesthesia induction and after MRI examination were 38.7 (37.6–39.3) and 38.2 (37.7–39.0) °C, respectively. Those of heat insulation group cats were 38.3 (37.6–39.5) and 37.5 (36.8–38.1) °C, respectively. Some dogs and cats in which the heat insulating device was used had a temperature below 37.5 °C, which is the first stage in hypothermia, but their body temperatures decreased more slowly than those in the control group.

## 4. Discussion

In this study, we found that bubble wrap and a down cloth blanket in the heat insulating group was effective in preventing a decrease in body temperature of dogs and cats under anesthesia during MRI exam.

In the preliminary study, the use of bubble wrap was effective in preventing a decrease in water temperature, as in previous studies on body temperature of animals and humans undergoing surgery [7,18]. The down cloth used in this study consisted of duck feathers; the ducks were waterfowl, and a study reported that waterfowl down plumage does not dissipate heat because it contains an abundance of air [21]. What determines heat retention is how much air can be stored, and the presence of a layer of air makes it difficult for heat to escape and contains warmth. Since down uses feathers as the material, it easily contains air and has high heat insulation. In addition, air has the lowest thermal conductivity compared to liquids and solids. The bubble wrap used contains air in cavities. Therefore, it was considered that the air layer of the two materials became a strong heat insulating material, preventing cold air penetrating from the outside and preventing body heat from escaping. The combined use of bubble wrap and down cloth was the most effective of the five conditions.

In this study, when the heat insulating group covered with the heat insulating device was compared to the control group not covered with the heat insulating device, the temperature differences immediately after the anesthesia induction and after the completion of the MRI exam were significantly less for both dogs and cats in the heat insulating group. In humans, the thermoregulatory mechanism is suppressed by the action of anesthetics [19,22,23]. Similar changes are expected to occur in dogs and cats [24]. The decrease in body temperature due to general anesthesia is classified into three stages: first phase (rapid phase), second phase (linear phase), and third phase (plateau phase). The first phase occurs within 1 h after anesthesia induction, and the redistribution of heat causes the central temperature to drop sharply by 1–1.5 °C. In the second phase, heat evaporation from the body surface to the outside increases. Furthermore, when the anesthesia time is 3 h or more and the amount of metabolic heat production becomes equal to the amount of heat loss, the third phase occurs, and the body temperature becomes constant. Considering that the MRI exam time is often approximately 30 min to 1 h, or, sometimes, 2 h, it is very important to prevent a decrease in body temperature in the first and second phases, that is preventing heat redistribution hypothermia and heat loss from the skin surface due to anesthesia. Heat redistribution hypothermia occurs after anesthesia induction, because the peripheral blood vessels rapidly dilate and heat diffuses throughout the body, resulting in a rapid decrease in the central temperature. Phase 2 heat loss is reported to be caused by four factors: radiation, evaporation, convection, and conduction of heat from animals [6]. The anesthetics used in this study were propofol for induction and isoflurane for maintenance. Since it was maintained with isoflurane during an MRI exam, it was considered that it was affected by isoflurane as one of the causes of the decrease in body temperature in this study. In this study, it was considered that using a heat insulating device to cover the entire body of the animal prevented heat diffusion. In addition, it was considered that the heat insulating device used in this study could protect the entire animal body from the outside air and minimize the heat loss from the body surface to the outside. A previous study reported that active warming of the limbs during surgery can prevent a drop in body temperature [14]. Sakata et al. [18] reported that active warming of the limbs during surgery and wrapping of a heat insulating device around the limbs and chests of cats that had undergone spay surgery helped prevent a drop in body temperature. Since animals are constantly exposed to the cold air of an MRI room, it is considered that covering the body with a heat insulation cover to prevent the body from touching the room air, like with the heat insulating device created in this study, helped prevent the body temperature from dropping. In this study, it was demonstrated that, by warming animals with a hot water bottle and preventing heat dissipation from the body with a heat insulating blanket, it was possible to moderate the decrease in body temperature in an environment with a low room temperature. There was no significant difference in the MRI exam times and body weights of the animals between the heat insulating group and the control group in this study. In addition to the drugs used and ASA classification, the duration of anesthesia is also a significant factor in the cause of hypothermia during anesthesia [25,26]. In addition, surface area per body weight has also been suggested to affect heat dissipation; a lighter body weight and larger body surface area per body weight contribute to a higher heat dissipation rate [27,28]. In this study, there was no significant difference in MRI exam time and body weight between the heat insulating group and the control group, suggesting that there was no effect of anesthesia time and body weight on body temperature changes. Jourdan et al. [29] examined the intravenous fluid line warmer in animals to prevent a drop in body temperature but reported that, if the administration rate and infusion line are long, the temperature of the warmed infusion drops before the infusion enters the body. This method cannot be used because a machine for warming the infusion solution is required and the room temperature in the MRI rooms is low, which may make it easier for the temperature of the infusion solution to drop. In human medicine, it is effective to perform prewarming before surgery and cover the body with a heat insulating device such as an aluminum sheet or bubble wrap [10,30,31]. In contrast, in veterinary medicine, the effectiveness of prewarming in dogs has not been shown [32,33], and the decrease in body temperature could not be suppressed during surgery by wrapping an aluminum sheet around a cat compared to when a warming device was applied on to the cat [13]. Either way, the use of aluminum sheets affects MRI images, making them difficult to apply to animals undergoing an MRI exam. Thus, there are various restrictions on attempts to prevent a decrease in body temperature in MRI room. The heat insulating device developed in this study was effective in preventing a decrease in body temperature of animals without requiring a special device or affecting the image. In addition, the heat insulating device produced in this study can be easily obtained. The down cloth can be washed when it gets dirty, and the bubble wrap that comes into direct contact with the animal’s body can be replaced after each use. The heat insulating device prepared in this study can be a useful tool for preventing the decrease in body temperature of animals during an MRI exam. A limitation of this study is that we did not compare the effects of “bubble wrap” and “down cloth” separately. It is possible that only one layer is effective to prevent drop in the body temperature. Additionally, a higher body surface to weight ratio contributes to a faster rate of heat dissipation. It is necessary to investigate the effects of body size in more detail, because we have used mostly small-breed dogs. Furthermore, in this study, both the control group and the heat insulation group installed a hot water bottle under the animal’s body, so it is unclear whether the heat insulation device is effective even without the hot water bottle. In addition, in this study, we investigated the change in body temperature from the induction of anesthesia to the end of MRI exam but did not investigate the time until the recovery of body temperature. Therefore, we would like to examine whether the heat insulating device designed in this study is helpful for recovering the body temperature that had dropped due to anesthesia.

## 5. Conclusions

In this study, it was clarified that a heat insulating bubble wrap and down cloth blanket that covered the limbs and the trunk were useful for preventing hypothermia during a head MRI exam of approximately 1 h.

## Figures and Tables

**Table 1:** Profiles of 24 dogs used in examination in this study.

No.	Breed	Age (Years)	Sex	Body Weight (kg)	Clinical Signs or Diagnosis	Group
1	French Bulldog	12	Male	12.6	Idiopathic epilepsy	Control
2	Shiba Inu	12	Spayed female	12.7	Meningioma	Control
3	Miniature Dachshund	9	Castrated male	5.0	Cushing syndrome	Control
4	Chihuahua	13	Male	2.2	Intranasal adenoma	Control
5	French Bulldog	11	Female	10.0	Pituitary adenoma	Control
6	Boston Terrier	10	Male	7.7	Pituitary adenoma	Control
7	Rough Collie	12	Male	24.5	Nasal discharge/epistaxis	Control
8	Toy Poodle	14	Castrated male	4.1	Rathke’s cleft cyst	Control
9	Mix	2	Spayed female	3.1	Necrotizing meningoencephalitis	Control
10	Pomeranian	13	Male	4.0	Nasal obstruction	Control
11	Mix	13	Castrated male	4.0	Cushing syndrome	Control
12	Boston Terrier	12	Spayed female	7.2	Chemodectoma	Control
13	Mix	5	Spayed female	5.2	Intranasal lymphoma	Control
14	Miniature Schnauzer	14	Spayed female	5.0	Meningioma	Control
15	Shiba Inu	12	Spayed female	14.6	Peripheral schwannoma	Control
16	French Bulldog	11	Castrated male	12.6	Glioma	Control
17	Shiba Inu	13	Male	11.4	Change to an aggressive character	Control
18	Pekingese	11	Spayed female	6.0	Idiopathic epilepsy	Heat insulating
19	Pembroke Welsh Corgi	12	Male	11.3	Pituitary adenoma	Heat insulating
20	Labrador Retriever	9	Female	35.5	Intranasal adenocarcinoma	Heat insulating
21	Papillon	12	Castrated male	3.9	Meningioma	Heat insulating
22	Miniature Schnauzer	12	Spayed female	7.0	Meningioma	Heat insulating
23	Mix	2	Spayed female	3.6	Necrotizing meningoencephalitis	Heat insulating
24	French Bulldog	9	Spayed female	10.0	Idiopathic epilepsy	Heat insulating

**Table 2:** Profiles of 26 cats used in examination in this study.

No.	Breed	Age (Years)	Sex	Body Weight (kg)	Clinical Signs or Diagnosis	Group
1	Mix	6	Spayed female	3.5	Nystagmus	Control
2	Mix	12	Castrated male	5.9	Intranasal adenocarcinoma	Control
3	Mix	13	Spayed female	5.2	Torticollis	Control
4	Mix	7	Castrated male	3.3	Nasal discharge/epistaxis	Control
5	Mix	3	Castrated male	3.5	Intranasal adenocarcinoma	Control
6	Mix	19	Castrated male	5.1	Pituitary adenoma	Control
7	Mix	6	Castrated male	4.0	Idiopathic epilepsy	Control
8	Mix	10	Spayed female	3.6	Intranasal adenocarcinoma	Control
9	Persian	8	Castrated male	3.1	Nasal discharge/epistaxis	Control
10	Mix	13	Castrated male	4.9	Soft tissue sarcoma	Control
11	Mix	8	Castrated male	5.2	Idiopathic epilepsy	Control
12	Mix	16	Castrated male	5.2	Pituitary adenoma	Control
13	Mix	15	Spayed female	2.4	Intranasal lymphoma	Control
14	Singapura	10	Castrated male	4.9	Intranasal lymphoma	Control
15	Mix	16	Castrated male	3.4	Intranasal adenocarcinoma	Heat insulating
16	Mix	6	Castrated male	5.6	Idiopathic epilepsy	Heat insulating
17	Mix	6	Castrated male	3.3	Idiopathic epilepsy	Heat insulating
18	Scottish Fold	18	Spayed female	3.9	Intranasal lymphoma	Heat insulating
19	Mix	15	Spayed female	3.6	Torticollis	Heat insulating
20	Mix	17	Castrated male	3.3	Intranasal lymphoma	Heat insulating
21	Mix	5	Castrated male	6.5	No clinical signs	Heat insulating
22	Mix	5	Castrated male	5.2	No clinical signs	Heat insulating
23	Sphynx	8	Spayed female	3.2	Intranasal adenocarcinoma	Heat insulating
24	Mix	6	Castrated male	4.8	No clinical signs	Heat insulating
25	Mix	5	Castrated male	5.0	No clinical signs	Heat insulating
26	Mix	5	Spayed female	3.7	Intranasal lymphoma	Heat insulating

## Data Availability

The data presented in this study are available on request from the corresponding author.
